# Gender difference in the association of coping styles and social support with psychological distress among patients with end-stage renal disease

**DOI:** 10.7717/peerj.8713

**Published:** 2020-03-26

**Authors:** Qi Wang, Hongjian Liu, Zheng Ren, Wenjing Xiong, Minfu He, Xinwen Fan, Xia Guo, Xiangrong Li, Hong Shi, Shuang Zha, Shuyin Qiao, Hanfang Zhao, Nan Li, Xiumin Zhang

**Affiliations:** 1 Department of Epidemiology and Biostatistics, School of Public Health, Jilin University, Changchun, China; 2 Department of Social Medicine and Health Management, School of Public Health, Jilin University, Changchun, China; 3 The First Hospital of Qiqihar City, Qiqihar, China

**Keywords:** Coping styles, End-stage renal disease, Psychological distress, Social support

## Abstract

**Objectives:**

The study aimed to explore the association of coping styles and social support with psychological distress among patients with end-stage renal disease (ESRD) as well as gender differences in these associations.

**Methods:**

A cross-sectional study of 769 ESRD patients was conducted at 25 hospitals in Qiqihar City, China. All information was collected using structured questionnaires that were self-administered by the patients. Patients’ psychological health status, coping styles and perceived social support were measured using the 12-item General Health Questionnaire, the Medical Coping Modes Questionnaire and the Multidimensional Scale of Perceived Social Support. Student’s *t* test, analysis of variance (ANOVA) and binary logistic regression analysis were used for data analysis.

**Results:**

A total of 72.3% of participants reported psychological distress, and the prevalence of distress was higher in females (77.0%) than in males (68.8%). The usage of the acceptance-resignation coping style was positively related to psychological distress (B = 0.459, *P* < 0.001). Social support level was negatively associated with psychological distress (B = −0.049, *P* < 0.001). The use of the confrontation style was negatively related to psychological distress in females (OR = 0.87, 95% CI [0.78–0.96]) but not in males (OR = 0.98, 95% CI [0.91–1.06]) (*P* for interaction term = 0.007).

**Conclusions:**

Greater use of the acceptance-resignation style and lower social support are related to a higher risk of psychological distress. Greater use of the confrontation style is related to a lower risk of psychological distress in females but not in males.

## Introduction

End-stage renal disease (ESRD) is the most advanced stage of chronic kidney disease (CKD), requiring life-sustaining renal replacement therapy, which consists of either dialysis or renal transplantation (Aileen et al., 2005). There is growing evidence for the high prevalence of psychological distress in ESRD patients, and such distress is associated with increased mortality, lower treatment compliance, and increased healthcare service utilization (Senanayake et al., 2018; Yagil et al., 2018; Joseph et al., 2011; Farrokhi et al., 2014). The reported prevalence of psychological distress in ESRD patients varies greatly, with estimates ranging from 25% to 75% (Senanayake et al., 2018; Ng et al., 2014; Dora, Lilla & Marta, 2012). ‘Psychological distress’ is often used as an indicator of emotional suffering. If protective strategies are not implemented, less serious but persistent psychological distress may develop into anxiety or depression (Kessler et al., 1997).

Based on the bio-psycho-social theory, psychological distress is considered as the final outcome developing from the interactions among multiple pathophysiological, psychological, and socioeconomic stressors affecting the individual (Dora, Lilla & Marta, 2012; Engel, 1981). Haemodialysis (HD) patients must face many possible stressors during the course of the disease, such as the impact of the unfavourable prognosis, lack of a curative treatment, physical deterioration, and increasing economic burden (Tu et al., 2014; Akin et al., 2010). ESRD patients experience impaired quality of life, and psychological problems have been identified as one of the major contributors to reduced quality of life in patients with ESRD.

Social support usually refers to the functions performed for the individual by three groups: family, friends and significant others (Zimet et al., 1990). Such support is helpful to improving patients’ psychological health. Coping is identified as the constant changes in action and cognition used to manage stressful demands (Lazarus & Folkman, 1984). In other words, when facing external or internal conflicts or demands, patients make cognitive or behavioural efforts to confront stressful situations. According to the stress-buffering model (Cohen & Wills, 1985), a positive coping style and a high level of social support offer buffering effects in the path of disease and psychological distress (Berkman, 1995). Therefore, as effective buffer variables, a positive coping style and a high level of social support may positively affect health outcomes and reduce psychological stress either directly or indirectly by buffering the negative effects when an individual encounters a disease.

Unlike for other patients, the constant threat of death, reduced life expectancy, decreased physical strength, and an intrusive medical regime have been shown to cause a higher incidence of psychological illness in dialysis patients than in other patients (Gilbar, Or-Han & Plivazky, 2005). Moreover, there are differences between male and female patients in the prevalence of psychological distress (Hettiarachchi & Abeysena, 2018). In addition, a previous study reported that men and women tended to have different coping strategies when faced with stress (Martin et al., 2013). One study (Pilar Matud, Concepcion Garcia & Fortes, 2019) reported that the levels of social support varied among men and women, which affected individuals’ mental health. Gender differences can affect the selection of coping styles and the social support level among ESRD patients, which may further affect their risk of experiencing psychological distress. Therefore, whether there are gender differences in the relationships of coping style and social support with psychological distress deserves examination. Psychological symptoms are common among haemodialysis patients (Chong & Tan, 2013). Research on gender differences in the effects of coping styles and social support on psychological distress can help physicians implement more targeted measures to reduce the risk of psychological distress in ESRD patients.

The objectives of this study were to explore the association of coping styles and social support with psychological distress and to ascertain gender differences in those associations. Knowledge of gender differences in coping styles and social support is helpful for doctors who are providing more personalized care, thereby enabling patients to obtain maximum health benefits from participating in rehabilitation. Based on the previous studies, the following hypotheses were tested:

(1) Based on the stress-buffering model (Cohen & Wills, 1985), a positive coping style and a high level of social support can buffer the negative effects encountered when an individual experiences a disease. We expected that greater use of the confrontation style and a higher level of social support would be associated with lower risk of psychological distress; in contrast, greater use of the avoidance or acceptance-resignation styles and a lower level of social support were associated with higher risk of psychological distress.

(2) Based on previous studies (Martin et al., 2013; Pilar Matud, Concepcion Garcia & Fortes, 2019), we predicted that gender may have a moderating effect on the associations of coping styles and social support with psychological distress among ESRD patients.

## Material and Methods

### Study design and population

This cross-sectional study was carried out in Qiqihar City, in northeastern China, from March to July of 2018. Twenty-five secondary and tertiary hospitals in Qiqihar City had haemodialysis qualifications during the investigation. We enrolled all eligible hospitals into the study. Patients met the inclusion and exclusion criteria and voluntarily participated in the study. Written informed consent was obtained from all patients before their participation in the survey. Participants completed a patient-reported questionnaire within 30 min after their haemodialysis treatment. Participants who expressed difficulty in completing the questionnaire answered questionnaire items verbally. Then, interviewer filled out the questionnaire. The questionnaire included general demographic variables, disease-related variables and several standardized structured scales. The investigators were trained doctors and nurses. We adhered to the bioethics principles of the Declaration of Helsinki and this study was approved by the Research Ethical Committee of Jilin University (Reference Number: 2017-12-18).

We recruited 870 patients to take the questionnaire survey, and 854 questionnaires were returned, with a response rate of 98.16%. Of these, 85 patients were excluded because they had other diseases that had a greater impact on their mental health statuses (e.g., cancer (Deimling et al., 2010), stroke (Hilari et al., 2010), and extremity disability (Kamlesh, Rajesh & Ajit, 2003)). The final sample size was 769 (443 males and 326 females, aged 18–94 years, M_age_ = 53.13, SD = 16.24) for the present analyses. The inclusion criteria were as follows: aged at least 18 years, both genders, with haemodialysis of at least three months, absence of cognitive dysfunction, understanding of own disease conditions, agreed to participate in the survey and signed the informed consent form. The exclusion criteria was any treatment for mental illness.

### Variables

Patients’ sociodemographic characteristics included age, gender, marital status (married and nonmarried), living situation (alone, with children, with spouse, and other) and monthly family income per capita (less than ¥2000, ¥2001–¥4000, and ¥4001 and above). The disease-related information consisted of caregiver (relatives, non-relatives), haemodialysis frequency (<3 times/week, 3 times/week, >3 times/week) and years on haemodialysis (<5 years, 5–10 years, >10 years). Patient psychological distress, coping styles and perceived social support were measured using the 12-item General Health Questionnaire (GHQ-12), the Medical Coping Modes Questionnaire (MCMQ) and the Multidimensional Scale of Perceived Social Support (MSPSS). Psychological distress was the outcome variable of the study. Gender, coping styles and social support were the expected explanatory variables. The remaining variables were covariates in the study.

### Instruments

#### The 12-item General Health Questionnaire (GHQ-12)

The Chinese version of the 12-item General Health Questionnaire (GHQ-12) was used to detect the level of general mental health among haemodialysis patients. This instrument was developed by Goldberg and revised by scholar Taian Cheng (Goldberg David & Williams, 1988; Cheng & Williams, 1986). The common method is binary GHQ scoring (0–0–1–1), which yields a possible score range of 0–12. The GHQ-12 includes six questions that are positively worded (e.g., Have you been able to concentrate on whatever you are doing?) and six that are negatively worded (e.g., Have you lost confidence in yourself?). The wording of the response scale is reversed for the two types of questions so that the responses for the positively worded questions range from 1 (never or occasionally) to 0 (sometimes or usually), and the responses for the negatively worded questions range from 0 (never or occasionally) to 1 (sometimes or usually). Higher scores indicate worse mental health. GHQ-12 scores of four and above indicate a tendency towards psychological distress (Goldberg et al., 2000). The coefficient of Cronbach’s *α* of this scale in the present study was 0.78.

#### Multidimensional Scale of Perceived Social Support (MSPSS)

Social support was measured by the Chinese version of the Multidimensional Scale of Perceived Social Support (MSPSS). The MSPSS is a 12-item instrument that measures perceived support from three sources: family (items3, 4, 8 and 11), friends (items 6, 7, 9 and 12) and significant others (items 1, 2, 5 and 10) (Zimet et al., 1988; Huang, Jin & Hong, 1996). Items are scored on a 7-point rating scale ranging from 1 (very strongly disagree) to 7 (very strongly agree), with possible total scores ranging from 12 to 84. Higher scores on each of the subscales indicate higher levels of perceived support, and the sum of the three subscales yields the perceived social support total score. The Chinese MSPSS has shown good reliability and validity in populations such as cancer patients, medical students and adolescents (Huang, Jin & Hong, 1996; Cheng & Chan, 2004; Chou, 2000). In the present study, the Cronbach’s *α* coefficient of MSPSS was 0.93.

#### Medical Coping Modes Questionnaire (MCMQ)

The Medical Coping Modes Questionnaire (MCMQ), originally conceived by Feifel, Strack & Nagy (1987), is a 19-item questionnaire that purports to measure the extent to which patients use three cognitive–behavioural coping strategies (confrontation, avoidance, and acceptance–resignation) in dealing with their illness. The MCMQ used in the present study is the Chinese version of the MCMQ, which had one item added during translation into Mandarin Chinese (Shen, 2000). Each item is scored on a 4-point Likert scale ranging from 1 to 4, and each score indicates a different frequency of each coping behaviour’s use (e.g., never, sometimes, frequently, all the time). Eight of the 20 items are reverse-scored. For each of the three ways of coping, a high score indicates the specific coping style that the patient often was used to deal with medical events. In the Chinese version of the MCMQ, the Cronbach’s *α* coefficients for the confrontation, avoidance, and acceptance–resignation subscales were 0.69, 0.60, and 0.76, respectively.

### Statistical analysis

Continuous variables were described as the mean and standard deviation when normally distributed and with median and interquartile range when the variables did not follow a normal distribution. Categorical variables were presented as absolute number (*n*) and relative frequency (%). Student’s *t* test or analysis of variance (ANOVA) was used to analyse the differences in coping styles and social support score distribution. Binary logistic regression analysis was used to explore the associations between psychological distress and selected factors, including age, gender, marital status, living situation, monthly family income per capita, caregiver, self-care ability, haemodialysis frequency, years on haemodialysis, coping styles and social support. Multivariate logistic regression analysis was used to examine associations between variables (gender, coping styles, social support and gender × social support/coping styles) and psychological distress. There were differences between male and female patients if the coefficient of the interaction term of gender × social support/coping styles was significant. Then, the interaction variables that were found to be significant were included into sex-stratified analysis to examine the gender difference.

Psychological distress identified by the GHQ-12 score was included as a dependent variable in such logistic regression models. We provided estimates of the associations by deriving odds ratios (ORs) and the corresponding 95% confidence intervals (CI) from the logistic regression models. To minimize multicollinearity, social support and coping styles were centralized (variable minus their mean) before the interaction terms were formed. The results of the tests were considered significant when the 2-sided *P* value was less than 0.05. All analyses were performed using IBM SPSS 24.0 (IBM Corp, Armonk, New York, USA).

**Table 1:** Descriptive statistics for coping styles and social support.

**Variables**	**Total(*n* = 769)**	**Confrontation**		**Avoidance**		**Acceptance–Resignation**		**Social support**	
		M (SD)	*P*	M (SD)	*P*	M (SD)	*P*	M (SD)	*P*
**Age, years**			0.688		**0.004**		0.257		0.118
≤49	325 (42.3)	19.80 (2.94)		16.78 (2.55)		12.24 (3.03)		55.99 (14.45)	
50–64	246 (32.0)	19.62 (2.94)		16.63 (2.68)		11.95 (2.85)		55.50 (13.72)	
≥65	198 (25.7)	19.61 (2.87)		16.01 (2.85)		12.40 (3.12)		58.07 (12.39)	
**Gender**			0.781		**0.002**		0.093		0.022
Male	443 (57.6)	19.72 (2.98)		16.79 (2.72)		12.03 (3.07)		57.34 (13.27)	
Female	326 (42.4)	19.66 (2.83)		16.18 (2.61)		12.40 (2.89)		55.05 (14.25)	
**Marital status**			0.539		0.158		0.890		**0.003**
Married	533 (69.3)	19.73 (2.92)		16.44 (2.63)		12.18 (3.06)		57.41 (12.80)	
Nonmarried	236 (30.7)	19.59 (2.91)		16.74 (2.81)		12.21 (2.86)		54.03 (15.41)	
**Living situation**			0.029		**0.001**		0.647		**<0.001**
Alone	166 (21.6)	19.75 (2.51)		17.16 (2.34)		12.45 (3.55)		49.18 (15.16)	
Live with children	90 (11.7)	18.88 (3.36)		15.80 (2.87)		12.19 (2.82)		60.19 (11.61)	
Live with spouse	470 (61.1)	19.85 (2.88)		16.48 (2.65)		12.12 (3.14)		57.87 (12.66)	
Other	43 (5.6)	19.37 (3.56)		16.26 (3.46)		12.00 (3.44)		59.67 (14.05)	
**Monthly family income per capita**			0.736		**<0.001**		0.328		**<0.001**
Less than ¥2000	336 (43.7)	19.60 (3.32)		16.18 (2.86)		12.36 (3.13)		57.29 (13.12)	
¥2001–¥4000	318 (41.3)	19.77 (2.59)		16.98 (2.30)		12.11 (2.82)		54.11 (14.63)	
¥4001 and above	115 (15.0)	19.73 (2.51)		16.37 (3.01)		11.91 (3.09)		59.94 (11.81)	
**Caregiver**			0.558		0.191		0.086		**<0.001**
Relatives	674 (87.6)	19.71 (2.93)		16.49 (2.70)		12.12 (3.04)		57.47 (12.98)	
Non-relatives	95 (12.4)	19.53 (2.83)		16.87 (2.59)		12.68 (2.67)		48.58 (16.27)	
**Self-care ability**			**<0.001**		0.096		**<0.001**		0.128
None	21 (2.7)	17.24 (3.97)		15.81 (4.07)		14.38 (3.15)		54.19 (18.08)	
Partial	266 (34.6)	19.79 (2.98)		16.32 (2.78)		13.12 (2.75)		55.15 (12.71)	
Full	482 (62.7)	19.74 (2.79)		16.68 (2.55)		11.58 (2.95)		57.13 (14.03)	
**Haemodialysis frequency**			0.590		**<0.001**		0.579		**<0.001**
<3 times/week	112 (14.6)	19.43 (2.10)		17.37 (2.32)		12.14 (2.20)		49.17 (16.95)	
3 times/week	646 (84.0)	19.74 (3.01)		16.42 (2.72)		12.21 (3.09)		57.49 (12.75)	
>3 times/week	11 (1.4)	19.73 (4.41)		14.91 (2.39)		11.27 (4.34)		63.73 (9.57)	
**Years on haemodialysis**			0.132		0.133		0.065		0.176
<5	505 (65.7)	19.70 (2.96)		16.57 (2.56)		12.02 (3.06)		56.22 (14.21)	
5–10	220 (28.6)	19.51 (2.79)		16.61 (2.93)		12.59 (2.84)		55.97 (12.58)	
>10	44 (5.7)	20.48 (2.96)		15.57 (2.81)		12.16 (3.00)		60.09 (13.42)	

**Notes.**

Differences were assessed using Student’s *t* test for gender, marital status and caregiver, and analysis of variance (ANOVA) for other variables. Bold values are statistically significant.

## Results

### Association of sociodemographic and clinical characteristics with coping styles and social support

A total of 769 patients aged 18–94 years were screened for the study, and the mean age of the participants was 53.13 ± 16.24 years. A total of 57.6% of the subjects were male. Based on the GHQ-12 scores, 556 ESRD patients (72.3%, 95% CI [69.1–75.5]) displayed psychological distress. The prevalence of psychological distress was marginally higher among males (68.8%; 95% CI [64.5–73.2]) than females (77.0%; 95% CI [72.4–81.6]), and the difference was statistically significant (*P* = 0.013). The differences in the demographic characteristics of patients who used confrontation, avoidance, acceptance–resignation and social support are summarized in Table 1. When patients lived with a spouse or engaged in partial self-care, they were more inclined to choose the specific coping style of confrontation. Male patients, those younger than 49 years old, living alone, with a monthly family income per capita of ¥2001–¥4000 or with a haemodialysis frequency less than 3 times/week were more inclined to choose avoidance. If patients could not care for themselves, they were more likely to choose acceptance–resignation. Married patients, male patients, those living with children, with a monthly family income per capita of ¥4001 and above, with a caregiver who was a relative, or with a haemodialysis frequency more than 3 times/week had higher social support scores than other patients (Table 1).

### Associations between psychological distress and selected factors

In identifying the associations between the selected factors and psychological distress, it was shown that psychological distress was significantly associated with gender, living situation, caregiver, self-care ability, haemodialysis frequency, coping styles and social support.

**Table 2:** Univariate logistic regression analyses of psychological distress.

**Variables**	**Psychological distress**		***P***	**OR(95% CI)**
	**No (*n* = 213)**	**Yes (*n* = 556)**		
**Age, years**^a^				
≤49	96 (29.5)	229 (70.5)	–	1.00
50–64	70 (28.5)	176 (71.5)	0.778	1.05 (0.73, 1.52)
≥65	47 (23.7)	151 (76.3)	0.150	1.35 (0.90, 2.02)
**Gender**^a^				
Male	138 (31.2)	305 (68.8)	–	1.00
Female	75 (23.0)	251 (77.0)	**0.013**	**1.51 (1.09, 2.10)**
**Marital status**^a^				
Married	152 (28.5)	381 (71.5)	–	1.00
Nonmarried	61 (25.8)	175 (74.2)	0.446	1.15 (0.81, 1.62)
**Living situation**^a^				
Alone	36 (21.7)	130 (78.3)	–	1.00
Live with children	24 (26.7)	66 (73.3)	0.370	0.76 (0.42, 1.38)
Live with spouse	140 (29.8)	330 (70.2)	**0.046**	**0.65 (0.43, 0.99)**
Other	13 (30.2)	30 (69.8)	0.241	0.64 (0.30, 1.35)
**Monthly family income per capita**^a^				
Less than ¥2000	91 (27.1)	245 (72.9)	–	1.00
¥2001–¥4000	83 (26.1)	235 (73.9)	0.776	1.05 (0.74, 1.49)
¥4001 and above	39 (33.9)	76 (66.1)	0.164	0.72 (0.46, 1.14)
**Caregiver**^a^				
Relatives	195 (28.9)	479 (71.1)	–	1.00
Nonrelatives	18 (18.9)	77 (81.1)	**0.044**	**1.74 (1.02, 2.99)**
**Self-care ability**^a^				
Full	172 (35.7)	310 (64.3)	–	1.00
None	1 (4.8)	20 (95.2)	**0.019**	**11.10 (1.48, 83.40)**
Partial	40 (15)	226 (85)	**<0.001**	**3.14 (2.13, 4.60)**
**Haemodialysis frequency**^a^				
3 times/week	194 (30)	452 (70)	–	1.00
<3 times/week	17 (15.2)	95 (84.8)	**0.002**	**2.40 (1.39, 4.13)**
>3 times/week	2 (18.2)	9 (81.8)	0.403	1.93 (0.41, 9.02)
**Years on haemodialysis**^a^				
<5	143 (28.3)	362 (71.7)	–	1.00
5–10	54 (24.5)	166 (75.5)	0.294	1.21 (0.85, 1.75)
>10	16 (36.4)	28 (63.6)	0.261	0.69 (0.36, 1.32)
**Coping styles**^b^				
Confrontation	20.10 (3.21)	19.53 (2.79)	**0.017**	**0.94 (0.89, 0.99)**
Avoidance	16.27 (2.90)	16.64 (2.60)	0.093	1.05 (0.99, 1.12)
Acceptance–Resignation	9.74 (2.70)	13.13 (2.54)	**<0.001**	**1.69 (1.55, 1.85)**
**Social Support**^b^	64.57 (10.01)	53.23 (13.67)	**<0.001**	**0.93 (0.91, 0.94)**

**Notes.**

a Categorical variables are presented as the frequencies and percentages.

b Normally distributed continuous variables are presented as mean and standard deviation.

Bold values are statistically significant.

**Table 3:** Multivariate logistic regression analysis of coping styles and social support on psychological distress.

**Variables**	**Model 1**		**Model 2**		**Model 3**	
	B	*P*	B	*P*	B	*P*
Confrontation	0.074	0.115	0.071	0.137	0.051	0.263
Avoidance	−0.019	0.707	0.009	0.873	0.038	0.364
Acceptance–Resignation	0.499	**<0.001**	0.499	**<0.001**	0.459	**<0.001**
Social Support	−0.062	**<0.001**	−0.065	**<0.001**	−0.049	**<0.001**
Gender (ref = male)	0.121	0.607	0.103	0.673	0.317	0.145
Gender × Confrontation	−0.24	**0.001**	−0.232	**0.003**	−0.201	**0.007**
Gender × Avoidance	0.091	0.264	0.095	0.271	–	–
Gender × Acceptance–Resignation	−0.098	0.305	−0.108	0.277	–	–
Gender × Social Support	0.031	0.086	0.035	0.067	–	–

**Notes.**

Model 1: Unadjusted. Model 2: Adjusted for age, marital status, living situation, monthly family income per capita, caregiver, self-care ability, haemodialysis frequency, and years on haemodialysis. Model 3: Variables of confrontation, avoidance, acceptance-resignation, social support, gender, gender × confrontation entered into the regression model. Adjusted for age, marital status, living situation, monthly family income per capita, caregiver, self-care ability, haemodialysis frequency, and years on haemodialysis.

Bold values are statistically significant.

The risk of psychological distress in female patients was 1.51 times as much as that for males (OR = 1.51, 95% CI [1.09–2.10]). Patients who lived with their spouse had lower risk of psychological distress (OR = 0.65, 95% CI [0.43–0.99]) than those living alone. The risk of psychological distress in patients who were cared for by non-relatives was higher than those cared for by relatives (OR = 1.74, 95% CI [1.02–2.99]). Compared with patients with normal self-care ability, patients without self-care ability and with partial self-care ability were 11.1 (OR = 11.1, 95% CI [1.48–83.40]) and 3.84 (OR = 3.84, 95% CI [2.30–6.40]) times as likely to experience psychological distress, respectively. The risk of psychological distress in patients with haemodialysis frequency less than 3 times/week was 2.4 (OR = 2.4, 95% CI [1.39–4.13]) times as much as patients with haemodialysis frequency equal to 3 times/week. Greater use of the acceptance-resignation style was closely related to a higher risk of psychological distress in patients (OR = 1.69, 95% CI [1.55–1.85]). Greater use of confrontation style was related to a lower risk of psychological distress in patients (OR = 0.94, 95% CI [0.89–0.99]). A higher social support score was associated with a lower risk of psychological distress (OR = 0.93, 95% CI [0.91–0.94]) (Table 2).

### The association of coping styles and social support with psychological distress

#### Coping styles

Table 3 presents the results of multivariate logistic regression analyses for the association of coping styles and social support with psychological distress. Model 1 was unadjusted, and models 2 and 3 were adjusted for covariates. The variables of confrontation, avoidance, acceptance-resignation, social support, gender, and gender × confrontation were entered into the regression model in model 3. For patients, acceptance–resignation demonstrated a positive association with psychological distress, indicating that patients who were more inclined to use the coping style of acceptance–resignation were more likely to experiencing psychological distress. The interaction of gender and acceptance–resignation was not significant (*P* = 0.277). However, the interaction of gender and confrontation was significant (*P* = 0.007), indicating that gender played a moderate role in the relationship between confrontation and psychological distress. This relationship was closer for females (*P* = 0.006) than for males (*P* = 0.585) (Table 4).

**Table 4:** Sex-stratified analysis of the association of confrontation with psychological distress.

**Variable**	**B**	**OR (95% CI)**	***P***
Male	−0.021	0.98 (0.91,1.06)	0.585
Female	−0.143	0.87 (0.78,0.96)	**0.006**

**Notes.**

Adjusted for age, marital status, living situation, monthly family income per capita, caregiver, self-care ability, haemodialysis frequency, and years on haemodialysis. Bold values are statistically significant.

#### Social support

For patients, social support demonstrated a negative association with psychological distress, indicating that patients who received less social support reported higher risk of psychological distress (B = −0.049, *P* < 0.001). In the regression analysis, association between the interaction term of gender × social support and psychological distress among all participants was not significant (*P* = 0.067). This result suggested that there were no gender differences in the association between social support and psychological distress in this study.

## Discussion

There were three major findings in this study. First, the overall prevalence of psychological distress in ESRD patients undergoing haemodialysis was high, and the prevalence of psychological distress was higher in females than in males. Second, coping style and social support were associated with psychological distress. Greater use of the confrontation style and a higher social support score were associated with lower risk of psychological distress. Greater use of the acceptance–resignation style and a lower score in social support were related to higher risk of psychological distress. Third, the association of acceptance-resignation and social support with psychological distress were independent of gender. There was a gender difference in the relationship between confrontation and psychological distress. Confrontation had a closer relationship with psychological distress in females than males.

Using the GHQ-12 questionnaire with a cut-off score of 4, the prevalence of psychological distress among the participants was found to be 72.3% (95% CI [69.1–75.5]). The percentage of psychological distress in ESRD patients was higher than in the general population (19%–28%) and in cancer patients (35%) (Stallman, 2010; Robinson, Mcbeth & Macfarlane, 2004; Zabora et al., 2001). Senanayake et al. (2018) reported a prevalence of psychological distress of 75% in patients with chronic renal failure. The main reason for this prevalence may have to do with haemodialysis being traumatic and chronic: it imposes substantial restrictions on patients and an economic burden on their families. The present study revealed that psychological distress was marginally more prevalent among females (77.0%; 95% CI [72.4–81.6]) than males (68.8%; 95% CI [64.5–73.2]). Some studies have shown that the prevalence of psychological distress in female patients was higher than that in male patients (Hettiarachchi & Abeysena, 2018). The present findings were compatible with those of other studies. Gender differences in psychological distress may be associated with sex-based social roles, emotional expression, coping style and social position (Tovt-Korshynska et al., 2001).

There were differences between male and female patients in coping styles and perceived social support scores. The coping style of avoidance was more commonly used by males than by females. The score for social support in male patients was higher than in female patients, which means that males may have a higher level of social support than females. Some studies have suggested that there are differences in social support between males and females (Shumaker & Hill, 1991; Neff & Karney, 2005; Depner & Ingersoll-Dayton, 1985). Depner & Ingersoll-Dayton (1985) found that females reported receiving less social support than males. The main reason for this discrepancy may be that social support is associated with the thinking model, perceptibility (Zhang et al., 2015) and demand (Dalgard et al., 2006). Shumaker & Hill (1991) suggested that gender differences in social support may be related to the social circle and healthy interpersonal relationships. For example, men’s socialization experiences and social networks are different from those of women.

A positive coping style and a high level of social support can buffer the negative effects when an individual encounters a disease. However, a greater use of the acceptance-resignation style was related to a higher risk of psychological distress, which confirmed hypothesis 1. The interaction of gender and acceptance–resignation was not significant, indicating that the association of the acceptance–resignation style with psychological distress was relatively independent of gender. The reason may be that, whether the patient is male or female, the acceptance–resignation style not only does not reduce the patient’s psychological stress but also increases the risk of psychological distress. Perceived social support may significantly influence psychological distress, not directly but as a buffer of the effect of disease (Kozora et al., 2005). Specifically, social support provided by families and friends allows patients to relieve stress, sustain individuals’ coping efforts and restore depleted psychological resources. The association between social support and psychological distress was not moderated by gender. Regardless of gender, social support may have similar functions in ESRD patients. First, such support helps distract their attention from the disease and provides some relaxation. Second, social support may be instrumental in providing help and reducing the burden of disease. Third, high levels of social support contribute to improving well-being and decreasing risk of psychological distress (Cramer, 1991; Wester et al., 2007).

The confrontation style was related to the low risk of psychological distress in females but not in males, which verified hypothesis 2. On the one hand, the reason for this relationship may be that there were differences in the stress resistance of different genders, and the same coping style had different influences on psychological health. Specifically, the prevalence of psychological distress was higher in females than in males. The relationship of the confrontation style with psychological distress was also closer for females than for males. On the other hand, the tendency to use the confrontation style was different between male and female patients. Although there was no significant difference in confrontation scores between males and females in this study, a previous study reported that the males were more inclined to cope actively with perceived stress than females (Joel, Atara Kaplan & Lchak, 1976). Men also regarded themselves as better able to cope with the physical aspects of illness (Lindqvist, Carlsson & Sj Dén, 2013).

Long-term haemodialysis treatment of ESRD patients will lead to complications, increased family financial burdens, gradual changes in social roles, etc., which can make patients prone to psychological distress. Coping style and social support can buffer the adverse impact of the disease on patients’ psychological health. Specifically, greater use of the positive coping style and a higher social support level are related to a lower risk of psychological distress. Medical staff and patients’ families should make efforts to improve patients’ social support level of patients. In addition, medical staff should guide patients to use the confrontation style to face their disease positively, especially for females. Cognitive-behavioural therapy (Teasdale et al., 1984) has supported that cognitive and behavioural interventions can relieve psychological illness. It is necessary to carry out guidance in coping styles and disease-related cognition for patients with psychological distress. For ESRD patients with psychological distress, coping and cognitive guidance can be used for early intervention in psychological symptoms.

Several limitations of this study should be considered. First, the design of this study was cross-sectional, and thus the results cannot be used to infer causal relationships. Second, all the information in our study stemmed from self-reported questionnaires completed by the participants; therefore, recall bias may exist in the process of information collection.

## Conclusions

The prevalence of psychological distress is high among ESRD patients undergoing haemodialysis, and it is higher in females than in males. Confrontation, acceptance-resignation and social support are associated with psychological distress. There is a gender difference in the association between confrontation and psychological distress. Confrontation has a closer relationship with psychological distress in females than males.

##  Supplemental Information

10.7717/peerj.8713/supp-1 Supplemental Information 1 Raw dataset (SAV format)

10.7717/peerj.8713/supp-2 Supplemental Information 2 Raw dataset (CSV format)

10.7717/peerj.8713/supp-3 Supplemental Information 3 Raw data key

## Additional Information and Declarations

### Data Availability

The following information was supplied regarding data availability: The raw data are available in the Supplemental Files.
