# Supplementary material for: Gender difference in the association of coping styles and social support with psychological distress among patients with end-stage renal disease
Source: PeerJ. 2020 Mar 26;8:e8713. doi: 10.7717/peerj.8713 (PMC7103200; doi:10.7717/peerj.8713)
Supplement: Supplemental Information 3 [file peerj-08-8713-s003.docx]

| Variables | Code | Code | Code | Code |
| --- | --- | --- | --- | --- |
| Gender | 1=“male” | 2=“female” |  |  |
| Age | 1=“≤49” | 2=“50~64” | 3=“≥65” |  |
| Marital status | 1=“Married” | 2=“Nonmarried” |  |  |
| Living situation | 1=“Alone” | 2=“Live with children” | 3=“Live with spouse” | 4=“Other” |
| Monthly family income per capita | 1=“≤2000” | 2=“2001~4000” | 3=“≥4000” |  |
| Caregiver | 0=“Relatives” | 1=“Nonrelatives” |  |  |
| Self-care ability | 1=“Full” | 2=“Partial” | 3=“None” |  |
| Years on hemodialysis | 1=“＜5” | 2=“5~10” | 3=“＞10” |  |
| Hemodialysis frequency | 1=“3 times” | 2=“＜3 times” | 3=“＞3 times” |  |
| GHQ12 Score (continuous) |  |  |  |  |
| Psychological distress | 0=“No” | 1=“Yes” |  |  |
| Confrontation (continuous) |  |  |  |  |
| Avoidance (continuous) |  |  |  |  |
| Acceptance–Resignation (continuous) |  |  |  |  |
| Social support score (continuous) |  |  |  |  |
| Centralized Confrontation (continuous) |  |  |  |  |
| Centralized Avoidance (continuous) |  |  |  |  |
| Centralized Acceptance-Resignation (continuous) |  |  |  |  |
| Centralized Social Support (continuous) |  |  |  |  |
